# Europa

**DOI:** 10.1007/s11577-020-00722-y

**Published:** 2020-11-24

**Authors:** M. Broll

**Affiliations:** Institut für Soziologie, Ludwig-Maximilians-Universität München, Konradstraße 6, 80801 München, Deutschland

## Gerhards, Jürgen, Holger Lengfeld, Zsófia S. Ignácz, Florian K. Kley und Maximilian Priem:

European Solidarity in Times of Crisis. Insights from a Thirteen-Country Survey. Abingdon/New York: Routledge 2019. 282 Seiten. ISBN 978-0-367-25728‑6. Preis: GBP 120,–.

Wie solidarisch ist Europa? Diese Frage stellt sich mit der Covid-19-Pandemie mit neuer Brisanz und womöglich aktueller denn je. Es geht dabei um die wechselseitige Übernahme von Intensivpatienten und -patientinnen, die Zusammenarbeit bei der Entwicklung eines Impfstoffes, jedoch auch um das anfängliche Zurückhalten von Schutzausrüstung und nationale Alleingänge und Schließungen. In den Verhandlungen über den Corona-Wiederaufbaufond der EU, der die ökonomischen und sozialen Folgen der Pandemie auffangen soll, wurden die verschiedenen Positionen zu Ausmaß und Konditionalität europäischer Solidarität deutlich. Während die deutsche Regierung von ihrer Position (auch im Vergleich zur letzten Wirtschaftskrise) abrückte und sich gemeinsam mit der französischen Regierung für ein umfassendes Hilfspaket aussprach, sind es die sogenannten sparsamen Vier (Österreich, die Niederlande, Dänemark und Schweden), die ihre Solidarität an starke Auflagen für die Empfänger knüpfen wollen. Einig sind sich die meisten Beteiligten jedoch darin, dass die gegenwärtige Krise und deren soziale Konsequenzen nur durch ein gemeinsames Vorgehen und europäische Solidarität abgefedert werden können. Dies wiederum setzt die Bereitschaft der Bevölkerungen in den Mitgliedsstaaten voraus.

Wie solidarisch ist also Europa? Diese Frage hat sich die Gruppe aus Forschern und Forscherinnen um Jürgen Gerhards, Holger Lengfeld, Zsófia S. Ignácz, Florian K. Kley und Maximilian Priem gefragt und zwischen Mai und November 2016 eine großangelegte Bevölkerungsumfrage („Transnational European Solidarity Survey-TESS“) in 13 europäischen Ländern durchgeführt, deren Ergebnisse nun in Buchform vorliegen. Sie bearbeiten ein Thema von hoher Aktualität und großer politischer und gesellschaftlicher Relevanz. Die Forschenden wollten bei ihrer Untersuchung wissen, wie groß die Zustimmung zu oder Ablehnung von europäischer Solidarität in den ausgewählten Ländern ist. Es geht somit nicht um solidarisches Handeln der befragten Menschen, sondern um deren *Solidaritätsbereitschaft*. Per Telefoninterview wurden jeweils 1000 Menschen in Deutschland, Frankreich, Griechenland, Irland, den Niederlanden, Österreich, Polen, Portugal, Schweden, Slowakei, Spanien und Ungarn und weitere 500 Menschen in Zypern befragt. Die Forschenden unterscheiden zwischen vier Formen institutionalisierter Solidarität: Fiskalische Solidarität im Sinne der Unterstützung von Mitgliedsstaaten in finanzieller Not; territoriale Solidarität als Umverteilung von Wohlstand von reicheren zu ärmeren Regionen; wohlfahrtsstaatliche Solidarität verstanden erstens als Unterstützung bedürftiger europäischer Bürger und Bürgerinnen, die von Erwerbslosigkeit, Alter oder Krankheit betroffen sind und zweitens als Redistribution von Wohlstand und der Abbau von sozialen Ungleichheiten. Die letzte Form institutionalisierter Solidarität ist die Binnen- und Außensolidarität hinsichtlich der Aufnahme und Verteilung von Geflüchteten. Die zugrundeliegende Annahme der Forschenden ist, dass die verschiedenen Krisen der vergangenen Jahre unterschiedliche solidarische Antworten beansprucht haben: Die Eurokrise ab dem Jahr 2008 erforderte fiskalische Solidarität. In der folgenden Wirtschaftskrise wurden territoriale und wohlfahrtsstaatliche Solidarität bedeutsam. Mit dem Sommer der Migration 2015 gelangte die Frage nach Solidarität mit Geflüchteten auf die Tagesordnung. Die Stärke der jeweiligen Form institutionalisierter, europäischer Solidarität wird in der Studie von Gerhards et al. nicht isoliert erhoben, sondern in Relation zu nationaler und globaler Solidarität bestimmt. Aus diesem Grund wurden ebenfalls die Einstellungen zu Solidarität in diesen anderen beiden räumlichen Bezugsgrößen ermittelt.

Die Forschungsergebnisse wurden jüngst bei Routledge unter dem Titel „European Solidarity in Times of Crisis: Insights from a Thirteen-Country Survey“ veröffentlicht. Das gut zu lesende Buch ist übersichtlich strukturiert, klar geschrieben und anschaulich illustriert. Einer Einleitung zu Krise und Solidarität in Europa folgt eine theoretische Konzeptualisierung des Begriffs der europäischen Solidarität. Im Zentrum des Buches stehen analog zu den vier Formen institutionalisierter Solidarität vier Kapitel, in denen die empirischen Ergebnisse dargelegt und diskutiert werden. Abschließend folgen eine Zusammenfassung der zentralen Ergebnisse sowie die Diskussion politischer Schlussfolgerungen.

Die Studienergebnisse überraschen in ihrer Deutlichkeit – nicht zuletzt angesichts der zähen Verhandlungen über die Corona-Bonds. Denn Gerhards et al. haben insgesamt sehr hohe Zustimmungswerte zu europäischer Solidarität unter den Befragten gefunden. So befürworten 66 % der Befragten fiskalische Solidarität in Europa, 71 % sind für eine Politik der territorialen Solidarität. Für wohlfahrtsstaatliche Solidarität sprechen sich zwischen 77 und 90 % der Befragten aus (abhängig davon, ob es um Unterstützung für Erwerbslose, Alte oder Kranke geht) und hinsichtlich der Solidarität mit Geflüchteten sind es in den ausgewählten Ländern durchschnittlich 81 %, die einer Gewährung des Bleiberechts zustimmen. In den einzelnen Ländern schwanken die Zustimmungsraten teilweise deutlich, dennoch liegen sie überall oberhalb der 50 Prozentmarke. Dies war für die Forscher und Forscherinnen ein Kriterium für die Existenz europäischer Solidarität.

Es stellt sich dennoch die (auch politische) Frage, wie die Diskrepanz zwischen hoher Solidaritätsbereitschaft einerseits und dem dieser Haltung widersprechenden Verhalten andererseits zu erklären ist. Warum wählen Menschen regelmäßig Parteien mit einer politischen Agenda, die ihren Einstellungen und Interessen zuwiderläuft? Das gilt nicht nur für europäische Politik, sondern muss ebenso für nationalstaatliche Solidarität diskutiert werden, für die die Zustimmungsraten noch höher sind. Und wer sich an den Diskurs in der sogenannten Griechenlandkrise erinnert, der mag sich zumindest über die deutlichen Zustimmungswerte zu einer solidarischen Politik in Europa wundern. Immerhin blieben damals hierzulande große Proteste gegen die auch von Deutschland forcierte europäische Austeritätspolitik aus, die maßgeblich dazu beigetragen hat, dass soziale Rechte in Griechenland abgebaut wurden und es zu massiven sozialen Verwerfungen kam.

Man könnte hier wie Jürgen Habermas in seiner Würdigung des Buches argumentieren und davon ausgehen, dass die politischen Eliten die Einstellungen ihrer Wählerschaft unterschätzen oder falsch auslegen. Auf der anderen Seite könnte man daran auch die Limitationen quantitativer Einstellungsforschung aufzeigen. Um die Ergebnisse etwas stärker zu kontrollieren, wurden die Teilnehmenden zwar ebenfalls dazu befragt, ob sie persönlich bereit wären die Kosten solidarischer Politik etwa durch Steuerabgaben mitzutragen. Auch hier stimmt eine Mehrheit (61 %) in den befragten Ländern zu. Dennoch bleibt notwendigerweise ungeklärt, wer dies auch tatsächlich tun würde und es nicht nur aus sozialer Erwünschtheit oder aufgrund der Unverbindlichkeit der Befragung angibt. Diese Problematik reflektieren die Autoren und Autorinnen teilweise im letzten Kapitel. Sich mit den Ergebnissen aber zu beruhigen und in ein Loblied auf europäische Solidarität zu verfallen, wäre mindestens naiv und würde das Handeln der Bevölkerungsmehrheiten nicht ernst nehmen, das im Wesentlichen aus schweigender Duldung oder aktiver Unterstützung von unsolidarischer Politik besteht. Die Diskrepanz zwischen den Befragungsergebnissen und der politischen Realität berührt daran anschließend die entscheidende Frage, wann eine solidarische Haltung in solidarische Praxis mündet. Denn nur sie allein vermag es, Europa solidarischer zu gestalten und Gesellschaft zu verändern.

